# Insecticidal Efficacy of *Satureja hortensis* L. and *Satureja khuzistanica* Jamzad Essential Oils Against *Callosobruchus maculatus* (F.)

**DOI:** 10.3390/plants14193062

**Published:** 2025-10-03

**Authors:** Asgar Ebadollahi, Bahram Naseri, Aysona Aghcheli, William N. Setzer

**Affiliations:** 1 Department of Plant Sciences, Moghan College of Agriculture and Natural Resources, University of Mohaghegh Ardabili, Ardabil 5697194781, Iran; 2 Department of Plant Protection, Faculty of Agriculture and Natural Resources, University of Mohaghegh Ardabili, Ardabil 5619911367, Iran; bnaseri@uma.ac.ir (B.N.); aysonaaghcheli@gmail.com (A.A.); 3 Department of Chemistry, University of Alabama in Huntsville, Huntsville, AL 35899, USA; 4 Aromatic Plant Research Center, 230 N 1200 E, Suite 100, Lehi, UT 84043, USA

**Keywords:** biopesticides, botanical agents, chemical profile, cosmopolitan insect pest, life-table parameters, HCA

## Abstract

The cowpea weevil, *Callosobruchus maculatus* (F.), stands out as one of the most destructive field-to-storage pests of leguminous crops. This study investigates the potential of essential oils derived from two *Satureja* species, *Satureja hortensis* L. and *Satureja khuzistanica* Jamzad, for managing *C. maculatus*. Bioassay results revealed that both *S. hortensis* (72 h LC_50_ = 0.20 µL/g) and *S. khuzistanica* (72 h LC_50_ = 0.19 µL/g) essential oils exhibited significant toxicity against *C. maculatus* adults. The essential oils extended development time, reduced adult longevity, and decreased fecundity of the pest. Key population parameters, including intrinsic growth rate (*r*) and net reproductive rate (*R*_0_), were significantly lowered, particularly by *S. hortensis* essential oil. Age-specific survival (*l_x_*) and fecundity (*m_x_*_)_ rates were also declined in treated groups, with delayed reproductive peaks compared to controls. Chemical analyses of *S. hortensis* and *S. khuzistanica* essential oils indicated that carvacrol (30.9% and 62.9%, respectively), γ-terpinene (25.5% and 4.3%), *p*-cymene (9.7% and 7.9%), and thymol (3.7% and 9.3%) were the major components. Hierarchical cluster analysis (HCA) was carried out to compare and contrast the compositions with previous works. The results demonstrated that *S. hortensis* and *S. khuzistanica* essential oils, given their lethal and sublethal effects against *C. maculatus*, can be introduced as natural alternatives to hazardous chemical insecticides, highlighting the need for further research in this field.

## 1. Introduction

The cowpea weevil, *Callosobruchus maculatus* (F.) (Coleoptera: Chrysomelidae), is a widely distributed insect pest that infests legumes, especially cowpea (*Vigna unguiculata* (L.) Walpers) seeds, both in the field and during storage [1]. As a cosmopolitan insect pest, *C*. *maculatus* is found in tropical and subtropical regions around the world and is originally native to Africa and Asia [2]. The feeding and activities of *C. maculatus* can cause significant quantitative and qualitative damage to crops, resulting in seed perforation, reduced marketability, decreased weight, and lower germination capacity [3].

The widespread use of chemical insecticides in the management of insect pests has resulted in several negative consequences, including potential risks to human health, environmental pollution, harm to non-target organisms, and the emergence of pest resistance [4,5]. For example, the resistance of *C. maculatus* to conventional pyrethroid and organophosphate insecticides, such as cypermethrin, permethrin, and pirimiphos-methyl has been reported [6]. Thus, the development of pest control agents that are both highly effective and environmentally benign, with minimal impact on non-target species, is crucial [7].

The genus *Satureja*, part of the Lamiaceae family, includes numerous species commonly known as savory [8]. Along with application in food industries, different biological effects of *Satureja* essential oils, from antioxidant to antibacterial and anticancer activities, were documented in the recent research [9,10,11]. Recent research has also shown that essential oils extracted from various species within this genus, which are rich in terpenes and phenylpropanoids, possess significant insecticidal properties for controlling insect pests [12,13,14]. For example, the essential oil from *Satureja hortensis* L., which is rich in the phenylpropanoid estragole (82.1%), demonstrated toxicity against adults of the lesser grain borer (*Rhyzopertha dominica* F.) and the red flour beetle (*Tribolium castaneum* Herbst), with 24 h LC_50_ values of 27.21 and 38.91 µL/L, respectively [15]. Additionally, the essential oil of *Satureja khuzistanica* Jamzad showed considerable insecticidal potential against fourth-instar larvae and adult Colorado potato beetles (*Leptinotarsa decemlineata* (Say)), with 24 h LC_50_ values of 23.36 ppm and 17.96 ppm, respectively [16]. The primary constituents of this essential oil were carvacrol (81.1%), *p*-cymene (3.3%), and γ-terpinene (3.2%).

The susceptibility of *C. maculatus* to plant-derived essential oils has been revealed in recent years [17,18]. More specifically, studies have examined not only acute lethal effects but also sublethal impacts of essential oils on biological and population parameters of *C. maculatus* [19,20,21,22]. Aimad et al. [21] reported substantial fumigant toxicity for three additional species, including wormwood (*Artemisia herba-alba* Asso), chamomile (*Matricaria recutita* L.), and yellow fleabane (*Dittrichia viscosa* L.). These treatments also reduced oviposition and emergence of adults. Notably, the essential oil of fennel (*Foeniculum vulgare* Mill.) significantly reduced biological (longevity, fecundity and oviposition period of adults) and key life table parameters (intrinsic rate of increase (*r*), net reproductive rate (*R*_0_), gross reproductive rate (*GRR*), and finite rate of increase (λ)) of the pest [14]. More precisely, the susceptibility of *C. maculatus* to the essential oils manifests not only in rapid pest mortality but also in significant alterations to its biological parameters and life table analysis. The evaluation of such parameters is a cornerstone of modern Integrated Pest Management (IPM), as it provides the essential quantitative data required to understand population dynamics, predict outbreak potential, and design effective, targeted control strategies [23,24]. Therefore, as part of eco-friendly management strategies for the *C. maculatus*, the present study was conducted to investigate the efficacy of essential oils extracted from two Iranian *Satureja* species, namely *S. hortensis* and *S. khuzistanica*, on the mortality and biological and demographic parameters of the pest. It should be emphasized that the toxicity of *S. khuzistanica* essential oil against *C. maculatus* has been investigated for the first time in the present study. Furthermore, the chemical composition of the studied essential oils was analyzed, and the potential relationship between the identified compounds and the observed insecticidal properties has been discussed.

## 2. Results

### 2.1. Chemical Profile of Commercial Essential Oils

A total of 46 compounds were identified in the essential oil of *S*. *hortensis*, accounting for 98.1% of the total composition, while 55 compounds were identified in *S*. *khuzistanica* essential oil, 97.5% of the total (Table 1). The major components in *S*. *hortensis* essential oil were carvacrol (30.9%), γ-terpinene (25.5%), and *p*-cymene (9.7%), while *S*. *khuzistanica* essential oil was dominated by carvacrol (62.9%), along with thymol (9.3%), and *p*-cymene (7.9%).

### 2.2. Acute Toxicity of Essential Oils

The results demonstrated that the tested concentrations of both *S. hortensis* (F = 30.79; df = 5, 71; *p* < 0.001) and *S. khuzistanica* (F = 59.72; df = 5, 71; *p* < 0.001) essential oils had significant effects on *C. maculatus* mortality. Similarly, exposure time significantly influenced mortality for both *S. hortensis* (F = 15.58; df = 2, 71; *p* < 0.001) and *S. khuzistanica* (F = 19.27; df = 2, 71; *p* < 0.001) essential oils. However, the interaction between exposure time and concentration was not significant for either *S. hortensis* (F = 0.26; df = 10, 71; *p* = 0.99) or *S. khuzistanica* (F = 0.35; df = 10, 71; *p* = 0.96).

The results of Probit analyses from lethality bioassays are presented in Table 2. The LC_50_ value for *S. hortensis* essential oil was 0.44 μL/g at 24 h, decreasing to 0.20 μL/g by 72 h. A similar trend was observed for *S. khuzistanica* essential oil, with LC_50_ values declining from 0.36 μL/g (24 h) to 0.19 μL/g (72 h). The proximity of the LC_50_ and LC_90_ values for all exposure times arises from the steep dose–response curve and the variability observed at high mortality levels. The lower LC_50_ values over time, coupled with higher relative potency, indicate that the toxicity of both essential oils increased significantly with prolonged exposure. Furthermore, R-squared (R^2^) values demonstrated a positive correlation between essential oil concentration and pest mortality, confirming concentration-dependent efficacy against *C. maculatus*.

### 2.3. Effects on Biological and Life Table Parameters

Effects of 24 h LC_30_ and LC_50_ values of *S. hortensis* (0.13 and 0.44 μL/g, respectively) and *S. khuzistanica* essential oils (0.15 and 0.36 μL/g, respectively) on the biological and life table parameters of *C. maculatus* are indicated at Table 3 and Table 4. The immature development (egg, larva and pupa period) of the pest was enlarged by the LC_50_ of *S. khuzistanica* essential oil. The immature survival has not shown a significant reduction with essential oils. The male adult longevity was shortened by *S. hortensis* essential oil, while the longevity of females was decreased by both essential oils and all treatments. Fecundity of the pest significantly decreased by both essential oils compared to the control group. The lowest fecundity was observed for adults treated with LC_50_ of *S. hortensis*. Total pre-ovipositional period (TPOP) was increased by the LC_50_ of *S. khuzistanica* essential oil (Table 3).

Intrinsic rate of population increase (*r*) and net reproductive rate (*R_0_*) were significantly decreased by the essential oil of *S. hortensis*. The *r* was also decreased by LC_50_ of *S. khuzistanica*. The gross reproductive rate (*GRR*) of the pest was significantly decreased by both essential oils compared to the control group. The finite rate of increase (*λ*) was also significantly decreased by the essential oil of *S. hortensis*. A diverse outcome was achieved for mean generation time (*T*): increasing by LC_50_ of *S. khuzistanica* and decreasing by LC_30_ of *S. hortensis* compared to the control group.

**Figure 1:**
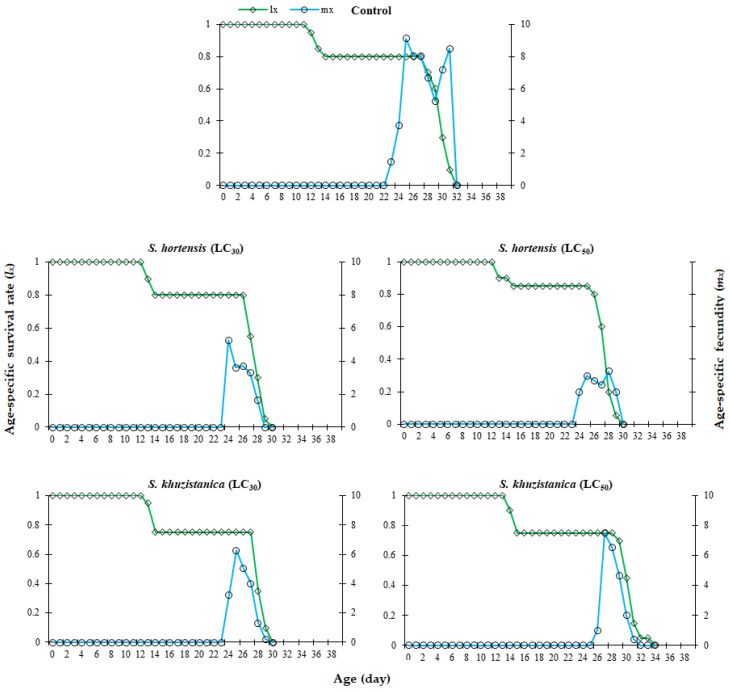
Age-specific survival rate (*l_x_*) and age-specific fecundity (*m_x_*) of *Callosobruchus maculatus* exposed to two different lethal concentration (LC) values of *Satureja hortensis* and *S. khuzistanica* essential oils compared to control responses.

Based on the age-specific survival rate (*l_x_*), the interval from first oviposition to death of the last female was 32 days in the control group. This duration was reduced to 30 days under treatment with LC_30_ and LC_50_ of *S. hortensis* essential oil, as well as the LC_30_ of *S. khuzistanica* essential oil. This reduction was accompanied by a noticeably smaller area under the *l_x_* curve, indicating decreased overall survival in treated populations compared to controls. The highest age-stage fecundity (*m_x_*) was observed in the control group (13.9 eggs at 25 days). Sublethal concentrations of *S. hortensis* essential oil significantly reduced *m_x_* to 5.25 eggs (at 24 days) for the LC_30_ treatment and 3.25 eggs (at 28 days) for the LC_50_ treatment. Similarly, exposure to *S. khuzistanica* essential oil resulted in *m_x_* of 6.25 eggs (at 25 days) and 7.50 eggs (at 27 days) for the LC_30_ and LC_50_ treatments, respectively. In general, *m_x_* of *C. maculatus* treated by both *Satureja* essential oils at all treatments was significantly decreased compared to the control group. These results indicate a concentration-dependent reduction in reproductive output in *C. maculatus*, with both essential oils demonstrating delayed peak fecundity compared to the control group (Figure 1).

## 3. Discussion

**Figure 2:**
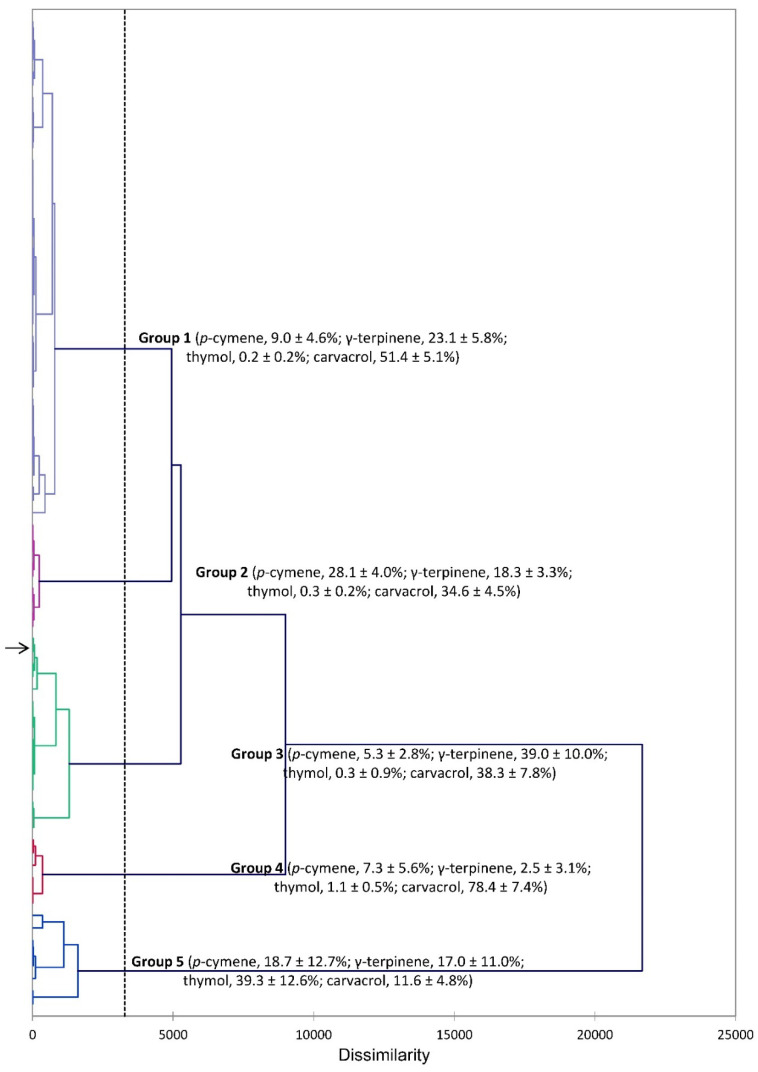
Dendrogram obtained from hierarchical cluster analysis (HCA) of *Satureja hortensis* essential oil compositions (major components). The essential oil sample from this study is indicated by the arrow (→).

The phytochemical profiles of *S. hortensis* were reviewed in 2018 [30], and there have been numerous publications on the essential oil compositions. The essential oils of *S. hortensis* are generally dominated by carvacrol, *p*-cymene, and γ-terpinene. In an analysis of 30 accessions of *S. hortensis* from Iran, two major clusters were identified: (1) carvacrol (42.0–58.2%), γ-terpinene (18.3–28.5%), and *p*-cymene (4.3–14.9%), and (2) dominated by carvacrol (76.0–83.3%) [31]. The chemical composition of an essential oil can have profound effects on biological activity. In order to place the composition of the essential oil used in this study with previous compositions, a hierarchical cluster analysis (HCA) was carried out. The HCA, based on the major essential oil components of essential oils from this work and those reported in the literature [9,32,33,34,35,36,37,38,39,40,41,42,43,44,45,46,47,48,49,50,51,52,53,54,55,56,57], reveals five major clusters (Figure 2): Group 1 is a carvacrol/γ-terpinene cluster, Group 2 is a carvacrol/*p*-cymene cluster. Group 3 has nearly equivalent concentrations of γ-terpinene and carvacrol. Group 4 has a high concentration of carvacrol. Group 5 has a high concentration of thymol. The essential oil of *S. hortensis* from this study is found in Group 3.

**Figure 3:**
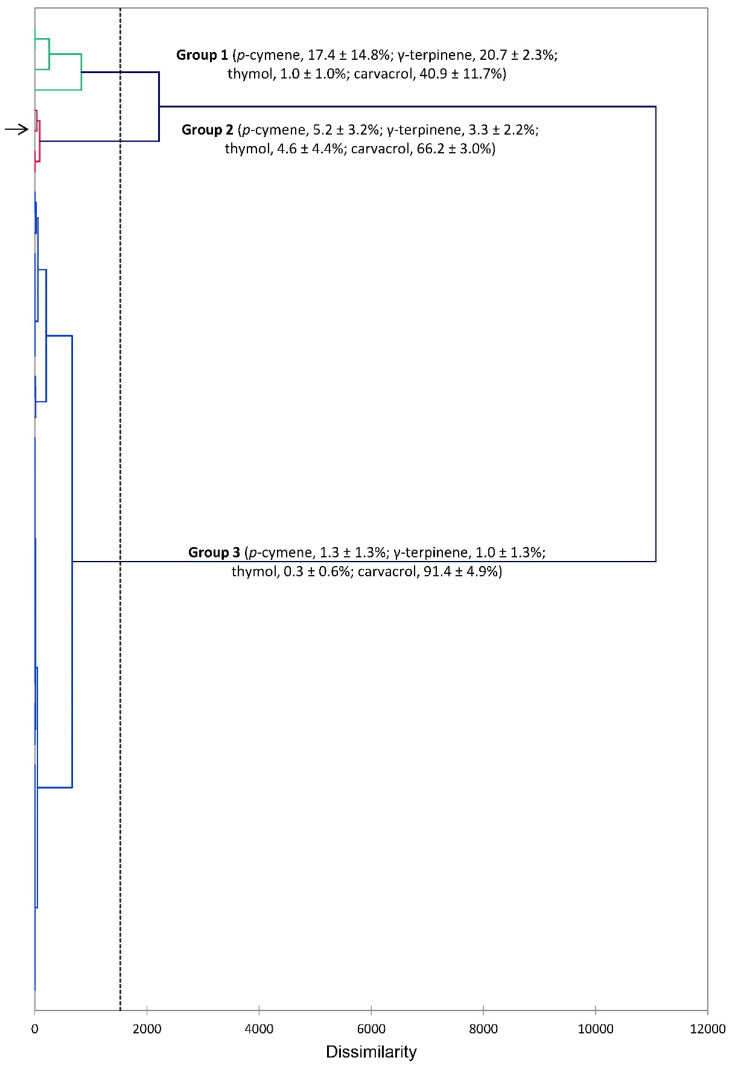
Dendrogram obtained from hierarchical cluster analysis (HCA) of *Satureja khuzistanica* essential oil compositions (major components). The essential oil sample from this study is indicated by the arrow (→).

The essential oils of *S. khuzistanica* are generally dominated by carvacrol. However, there is some variation with some samples showing lower levels of carvacrol. An HCA treatment based on the compositions of the essential oils from this study and those previously reported [38,58,59,60,61,62,63,64,65,66,67,68,69,70,71,72,73,74,75,76,77,78,79,80,81,82,83,84,85,86] illustrates these variations (Figure 3). The HCA shows three groups, which are separated based on the concentrations of carvacrol. Group 1 shows relatively low concentrations of carvacrol (40.9 ± 11.7%), Group 2 has moderate levels of carvacrol (66.2 ± 3.0%), and Group 3, the largest group, has high concentrations of carvacrol (91.4 ± 4.9%). The *S. khuzistanica* sample from this present study is found in Group 2.

The essential oils of *S. hortensis* and *S. khuzistanica* demonstrated significant efficacy against *C. maculatus*, with mortality rates increasing over time. While the insecticidal potential of *S. khuzistanica* essential oil against *C. maculatus* is reported here for the first time, the toxicity of *S. hortensis* essential oil has been documented in previous studies, which corroborate our findings. For instance, Heydarzade and Moravvej [12] reported contact toxicity of *S. hortensis* oil (sourced from the Mashhad province of Iran), with LC_50_ values of 535.7 and 641.0 μL/m^2^ against male and female adults of *C. maculatus*, respectively. The major constituents of essential oil were carvacrol (50.1%), thymol (27.8%), γ-terpinene (4.7%), and *p*-cymene (4.3%). Although these compounds were also identified in our study, their quantity differed considerably: 30.9%, 3.7%, 25.5%, and 9.7%, respectively. In another study, Zandi-Sohani [87] found that *S. hortensis* essential oil (from the Khuzestan province of Iran) had an LC_50_ value of 1.50 μL/L against *C. maculatus* adults via fumigation, with a concentration of 60 μL/L causing 91.1% mortality after 24 h. Zandi-Sohani [87] used filter paper fumigation bioassays, while our treatment involved impregnating cowpea seeds with the essential oil, a method that combines contact and fumigant actions and is highly relevant for practical pest management in field and storage conditions. Accordingly, the disparity in LC_50_ values between our study (0.44 μL/g) and those mentioned above can be attributed to the geographical origin of the plant material, variations in essential oil chemical composition, and differences in bioassay methodologies. Furthermore, the insecticidal effects of dominant terpenes identified in *S. hortensis* and *S. khuzistanica* essential oils, including carvacrol, *p*-cymene, thymol, and γ-terpinene, are well-documented in previous studies [47,88,89]. Additionally, recent studies have elucidated the multi-faceted modes of action exhibited by plant-derived compounds such as carvacrol, thymol, and γ-terpinene against insect pests. For instance, thymol application in the cotton bollworm (*Helicoverpa armigera* (Hübner)), was found to suppress key detoxification enzymes, including general esterases, glutathione *S*-transferase, and cytochrome P450, as well as inhibit acetylcholinesterase activity [90]. A similar inhibitory effect was observed in the red flour beetle (*Tribolium castaneum* Herbest), where carvacrol exposure led to decreased activity of both glutathione *S*-transferase and acetylcholinesterase [91]. Immunosuppressive properties and potential for genome damage of γ-terpinene against the melon fruit fly (*Zeugodacus cucurbitae* (Coquillett)) were also revealed [92]. Interestingly, the essential oil of *S. hortensis* (γ-terpinene/carvacrol-rich chemotype) was generally more active than the essential oil of *S. khuzistanica* (4.3% γ-terpinene, 62.9% carvacrol). The dual activities of γ-terpinene and carvacrol may be responsible for the more pronounced activity of *S. hortensis* essential oil. It can therefore be concluded that the insecticidal efficacy of *S. hortensis* and *S. khuzistanica* essential oils is likely due to the presence and multiple action of these compounds in their essential oils.

Along with lethality, the essential oils of *S. hortensis* and *S. khuzistanica* disrupted the life history parameters of *C. maculatus*. Treatment with *S. khuzistanica* essential oil resulted in increased developmental duration, a longer total pre-ovipositional period, and decreased immature survival. In general, exposure to sublethal stressors similar insecticide treatments can prolong developmental time and reduce survival rates [14,93]. Both essential oils significantly reduced the female longevity and fecundity of *C. maculatus* compared to the control. The lowest fecundity, representing a 78% reduction relative to the control, was observed in adults treated with the *S. hortensis* essential oil.

The reduction in fecundity may be attributed to several factors, including adult female mortality, decreased oviposition, and disruption of the vitellogenesis process [14,94,95]. The disruption of *C. maculatus* biological parameters by essential oils is consistent with previous research. For instance, Elhourri et al. [95] reported that the essential oil of *Chenopodium ambrosioïdes* L. decreased the survival, adult longevity, and fecundity of *C. maculatus*. Similarly, essential oils from *Majorana hortensis* Moench., *Rosmarinus officinalis* L., *Syzygium aromaticum*, and *Thymus vulgaris* L. negatively affected its fecundity [96]. A recent study by Naseri et al. [14] aligns with our findings, showing that essential oils of *Foeniculum vulgare* Mill. and *Pimpinella anisum* L. reduced immature survival, adult longevity, fecundity, and oviposition period. Furthermore, the essential oil of *S. hortensis* and its main component, carvacrol (the dominant compound in both essential oils studied here), reduced the emergence and longevity of the gray knot-horn (*Acrobasis advenella* (Zinck.)) [47]. Collectively, these results confirm that plant essential oils can alter the biological parameters of *C. maculatus*, an effect likely mediated by their specific chemical constituents.

The present study demonstrated that the essential oils of *S. hortensis* and *S. khuzistanica* significantly affected key life table parameters of *C. maculatus*, including the intrinsic rate of increase (*r*), net reproductive rate (*R*_0_), gross reproductive rate (*GRR*), finite rate of increase (*λ*), and mean generation time (*T*). Although reductions in these demographic indices have been previously documented for other essential oils [14,97,98], the effectiveness of *S. hortensis* and *S. khuzistanica* essential oils is reported here for the first time. These findings show that, besides their acute lethal effects, both essential oils can significantly reduce the population growth of *C*. *maculatus* in next generations, validating their potential as effective natural insecticides.

## 4. Materials and Methods

### 4.1. Essential Oils and Their Chemical Profile

The essential oils of *S. hortensis* and *S. khuzistanica* (100% purity) were obtained from Barij Essence Company (Kashan, Esfahan, Iran). The essential oils were stored at 4 °C in a refrigerator until use. The chemical profile of essential oils was analyzed using gas chromatography (Agilent 7890B, Santa Clara, CA, USA) coupled with a mass spectrometer (Agilent 5977A, Santa Clara, CA, USA). The evaluation was performed using an HP-5 ms capillary column (30 m × 0.25 mm × 0.25 µm). The carrier gas was helium with a column head pressure of 8.23 psi (56.8 kPa) and a flow rate of 1.0 mL/min. Inlet temperature was 280 °C and interface temperature was 280 °C. The GC oven temperature program was used as follows: 60 °C initial temperature, hold for 5 min; increased at 6 °C/min to 240 °C, then 10 °C/min to 310 °C. A 1% *w*/*v* solution of the sample in methanol was prepared and 1 μL was injected using a split ratio of 100:1. Identification of the oil components was based on their retention indices, determined by reference to a homologous series of *n*-alkanes [99], and by comparison of their mass spectral fragmentation patterns with those reported in the databases [25,26,27,28].

### 4.2. Insect Pest Rearing

The initial population of *C. maculatus* was sourced from a laboratory colony maintained on mung bean (*Vigna radiata* (L.) at the University of Mohaghegh Ardabili, Iran. For colony maintenance, 200 g of mung bean seeds (*Vigna radiata* (L.) ‘Parto’ cultivar) were placed in wide-mouthed cylindrical five-glass jars (18.5 cm diameter × 8 cm height) covered with mesh fabric to ensure proper ventilation. Each jar was infested with 50 randomly selected adult insects of mixed sex, and the jars were kept in a growth chamber under controlled conditions of 28 ± 1 °C, 60 ± 5% relative humidity, and a photoperiod of 14 h of light and 10 h of darkness. Newly emerged adults, 24 h old, from the colony were used for all bioassays [100].

### 4.3. Acute Toxicity of Essential Oils

Initial experiments were conducted to determine the minimum and maximum concentrations of essential oils needed to achieve mortality rates of approximately 25% to 75%. Based on logarithmic intervals, six concentrations were selected for the main bioassays, along with a control: 1.0–17.0 and 1.0–12.0 μL/7 g for *S. hortensis* and *S. khuzistanica* corresponding to 0.14–2.43 and 0.14–1.71 μL/g, respectively. For each treatment, the concentration was mixed with 2 mL of acetone and applied to 7 g of green gram seeds in a glass Petri dish (8 cm in diameter and 2 cm in height). The mixture was stirred thoroughly with a metal spoon for 2 min at room temperature until it was completely dry. The control treatment consisted of seeds treated with acetone alone, without any essential oil. After drying, the treated seeds were transferred into 6 cm Petri dishes, where 10 adults (5 males and 5 females) were introduced into each dish. Each treatment was replicated four times, and mortality was assessed at 24, 48, and 72 h post-treatment. Insects were considered dead if they showed no movement in their legs or antennae when gently prodded with a brush [14].

### 4.4. Effects on Biological and Life Table Parameters

The effects of 24 h LC_30_ and LC_50_ values of *S. hortensis* (0.13 and 0.44 μL/g, respectively) and *S. khuzistanica* (0.15 and 0.36 μL/g, respectively) essential oils on life history parameters of *C. maculatus* were evaluated. Eighty 1-day-old weevils (40 males + 40 females) of were exposed essential oils, while the control group was treated only with the solvent acetone. After 24 h, adult insects were removed from the rearing environment, and individual seeds containing single eggs were transferred to 6 cm diameter Petri dishes covered with mesh fabric. The seeds were monitored daily, and the emergence time of each adult insect was recorded. Upon female emergence, they were separately paired with male insects from the same treatment group. Adult longevity was recorded until the death of the last surviving male and female individuals. All procedures, except for the treatment of adult insects with the essential oils, were also performed on the control group and the collected data were subsequently analyzed to evaluate key biological parameters of the pest population, including pre-adults longevity and survival and adult longevity and fecundity. The age-stage-specific survival rate (*l_x_*; probability of surviving to age x) and age-stage-specific fecundity (*m_x_*; mean number of eggs laid per individual at age x) were calculated to estimate life table parameters [101,102]. These variables were computed using the following equations (where *x* = age, *j* = stage, and *n* = total number of developmental stages):

$$l_{x}=\sum_{j=1}^{n}s_{xj}$$

$$m_{x}=\sum_{j=1}^{n}s_{xj}f_{xj}/\sum_{j=1}^{n}s_{xj}$$

The intrinsic rate of increase (*r*), net reproductive rate (*R*_0_), gross reproductive rate (*GRR*), finite rate of increase (*λ*), and mean generation time (*T*) were also computed by the following formula:

$$\sum_{x=0}^{\infty }{e^{-r\;(x+1)}\;l}_{x}m_{x}=1$$

$$R_{0}=\sum_{x=0}^{\infty }l_{x}m_{x}$$

$$GRR=\sum_{x=0}^{\infty }m_{x}$$

*λ* = e*^r^*

*T* = (Ln *R*_0_)/*r*

### 4.5. Statistical Analyses

The acute toxicity data were subjected to analysis of variance (ANOVA), with mean comparisons performed using the Tukey HSD test (*p* < 0.05). Bioassay data for lethal concentration estimation and regression line analysis were analyzed using Probit analysis in SPSS software (Version 16). Life table parameters were analyzed using the TWOSEX-MSChart software (Version 21/10/2023) [103], with 100,000 bootstrap repetitions employed. Statistical differences between biological parameters were determined using paired bootstrap tests (*p* < 0.05). Agglomerative hierarchical cluster analyses (HCA) were carried using XLSTAT v. 2018.1.1.62926 (Addinsoft, Paris, France). In both *S. hortensis* and *S. khuzistanica*, the percentages of four components (*p*-cymene, γ-terpinene, thymol, and carvacrol) were used for the analysis. Dissimilarity was used to determine clustering based on Euclidean distance, and Ward’s method was used to define agglomeration.

## 5. Conclusions

Based on the present findings, the essential oils of *S. hortensis* and *S. khuzistanica* exhibited significant toxicity against adult *C. maculatus*, with lethality increasing over time. In addition to these acute lethal effects, application of the oils at their LC_30_ and LC_50_ caused significant disruption to several biological parameters of the pest, including prolonged larval and pupal development, reduced immature survival, shortened adult longevity, a decreased oviposition period, and a 54–78% reduction in fecundity. Furthermore, key population growth parameters *r*, *R*_0_, *GRR*, and *λ* were all significantly reduced compared to the control group. This indicates a strong inhibitory effect of the essential oils on the population growth of the subsequent generation. Chemical analyses identified terpenes as the dominant compounds in both oils, primarily monoterpene hydrocarbons and oxygenated monoterpenoids such as carvacrol, γ-terpinene, *p*-cymene, and thymol. In conclusion, the terpene-rich essential oils of *S. hortensis* and *S. khuzistanica* show great potential as accessible, effective, and eco-friendly botanical insecticides for the management of *C. maculatus*. In the present study, both contact and fumigant effects of the essential oils contributed concurrently to the observed mortality of the pest. Given that *C. maculatus* is a field-to-storage pest, separate investigations into contact and fumigant toxicity would be highly beneficial for potential field applications and direct protection of stored grains, respectively. To facilitate their practical application, further research is highly recommended in the following areas: evaluation of insecticidal activities of essential oils from other *S. hortensis* and *S. khuzistanica* chemotypes, investigating the side effects on non-target organisms (particularly beneficial predators and parasitoids), enhancing stability and persistence through advanced formulation technologies like micro- and nano-emulsions, and evaluating the residual effects of these essential oils on treated stored seeds.

## Figures and Tables

**Table 1:** Chemical profile and percent composition of essential oils isolated from *Satureja hortensis* and *S*. *khuzistanica*.

RI_calc_	RI_db_	Compound	Percent Composition	
			*S. hortensis*	*S. khuzistanica*
839	837	Furfural	-	tr
851	842	Ethyl 2-methylbutyrate	-	0.4
923	924	α-Thujene	0.4	tr
929	933	α-Pinene	3.5	1.2
942	950	Camphene	-	0.3
977	974	β-Pinene	0.7	0.1
981	982	1-Octen-3-ol	0.2	0.1
991	988	Myrcene	2.4	1.7
1003	1002	α-Phellandrene	0.3	0.2
1013	1008	δ-3-Carene	-	0.2
1016	1014	α-Terpinene	3.9	1.4
1026	1025	*p*-Cymene	9.7	7.9
1028	1030	Limonene	1.9	-
1028	1031	β-Phellandrene	-	0.2
1030	1032	1,8-Cineole	1.9	-
1062	1057	γ-Terpinene	25.5	4.3
1086	1086	Terpinolene	0.5	0.5
1104	1101	Linalool	-	0.7
1116	1113	*p*-Mentha-1,3,8-triene	-	tr
1165	1172	Borneol	0.2	0.1
1174	1174	Terpinen-4-ol	1.5	0.9
1185	1186	α-Terpineol	0.2	0.5
1197	1195	Methyl chavicol (=Estragol)	0.4	-
1236	1239	Thymyl methyl ether	0.1	-
1241	1238	Cuminal	0.3	-
1243	1244	Carvacryl methyl ether	0.9	0.5
1288	1289	Thymol	3.7	9.3
1298	1298	Carvacrol	30.9	62.9
1356	1356	Eugenol	0.1	0.1
1369	1365	Carvacryl acetate	0.1	0.4
1384	1380	(*E*)-β-Damascenone	0.1	-
1422	1424	(*E*)-β-Caryophyllene	2.1	0.5
1433	1432	*trans*-α-Bergamotene	tr	0.1
1440	1439	Aromadendrene	0.4	-
1448	1447	Geranyl acetone	tr	0.1
1452	1454	(*E*)-β-Farnesene	tr	tr
1455	1454	α-Humulene	0.1	tr
1462	1458	Alloaromadendrene	0.1	-
1477	1478	γ-Curcumene	-	tr
1480	1482	*ar*-Curcumene	-	tr
1485	1486	Phenylethyl 2-methyl butanoate	-	tr
1489	1490	Phenylethyl 3-methyl butanoate	-	tr
1496	1491	Viridiflorene	0.4	-
1500	1501	(*Z*)-α-Bisabolene	-	0.1
1505	1505	(*E*,*E*)-α-Farnesene	-	0.4
1507	1505	β-Bisabolene	1.9	1.6
1513	1511	Sesquicineole	-	tr
1521	1521	β-Sesquiphellandrene	-	tr
1532	1524	Dihydroactinidiolide	-	tr
1539	1541	(*E*)-α-Bisabolene	0.2	0.3
1558	1555	Elemicin	-	tr
1580	1578	Spathulenol	1.0	-
1587	1587	Caryophyllene oxide	0.5	0.2
1594	1600	Rosifoliol	-	0.1
1611	1608	Humulene epoxide II	-	tr
1634	1630	γ-Eudesmol	-	tr
1640	1639	Caryophylla-4(12),8(13)-dien-5β-ol	-	tr
1641	1640	τ-Cadinol	tr	-
1643	1656	Valerianol	-	tr
1669	1668	14-Hydroxy-9-epi-(*E*)-caryophyllene	0.1	0.1
1675	1671	β-Bisabolol	-	tr
1679	1679	*epi*-α-Bisabolol	tr	0.1
1739	1729	*iso*-Bicyclogermacrenal	0.1	-
1839	1841	Phytone	0.3	0.1
1920	1921	Methyl palmitate	0.1	-
1924	1916	(5*E*,9*E*)-Farnesyl acetone	-	tr
1960	1958	Palmitic acid	1.0	-
2002	2000	9β-Isopimara-7,15-diene	0.1	-
2050	2046	Kaur-16-ene	-	tr
2108	2109	Phytol	0.2	tr
2135	2134	Linolenic acid	0.1	-
		Monoterpene hydrocarbons	48.9	18.2
		Oxygenated monoterpenoids	39.7	75.2
		Sesquiterpene hydrocarbons	5.2	2.9
		Oxygenated sesquiterpenoids	1.6	0.5
		Diterpenoids	0.3	tr
		Benzenoid aromatics	0.6	0.1
		Others	1.8	0.6
		Total identified	98.1	97.5

RI_calc_ = Retention index calculated with respect to a homologous series of *n*-alkanes on a HP-5ms column. RI_db_ = Retention index from the databases [25,26,27,28].

**Table 2:** Probit analyses of the mortality of *Callosobruchus maculatus* adults exposed to *Satureja hortensis* and *S. khuzistanica* essential oils after 24 and 48, and 72 h.

Essential Oil	Time (h)	Lethal Concentrations with 95% Confidence Limits (μL/g)			x^2^ (*df* = 4)	Slope ± SE	Sig.	R^2^	RP
		LC_30_	LC_50_	LC_90_					
*S. hortensis*	24	0.13 (0.04–0.22)	0.44 (0.27–0.64)	8.31 (3.65–51.83)	0.66	1.00 ± 0.20	0.96	0.97	1.00
	48	0.12 (0.06–0.18)	0.26 (0.18–0.35)	1.91 (1.28–3.74)	0.60	1.49 ± 0.23	0.96	0.99	1.69
	72	0.11 (0.07–0.16)	0.20 (0.14–0.26)	0.83 (0.63–1.25)	1.54	2.09 ± 0.30	0.76	0.97	2.20
*S. khuzistanica*	24	0.15 (0.07–0.22)	0.36 (0.25–0.47)	3.18 (1.83–9.34)	0.19	1.35 ± 0.24	0.10	0.99	1.22
	48	0.12 (0.07–0.27)	0.24 (0.17–0.31)	1.30 (0.92–2.28)	0.21	1.75 ± 0.27	0.10	0.99	1.83
	72	0.10 (0.05–0.14)	0.19 (0.13–0.24)	0.90 (0.67–1.44)	1.27	1.88 ± 0.29	0.87	0.97	2.32

Sig., R^2^ and RP are Significant, R-squared and Relative Potency, respectively. Since the significant level is greater than 0.05, no heterogeneity factor is used in the calculation of confidence limits. Relative Potency = (the most LC_50_ value)/(LC_50_ value of the other essential oil) [29].

**Table 3:** Biological parameters (mean ± SE) of *Callosobruchus maculatus* treated with *Satureja hortensis* and *S. khuzistanica* essential oils and on untreated control group.

Parameter	Control	*S. hortensis*		*S. khuzistanica*	
		LC_30_	LC_50_	LC_30_	LC_50_
Development period (day)	24.37 ± 0.15 b	24.37 ± 0.12 b	24.41 ± 0.12 b	24.66 ± 0.12 b	26.8 ± 0.19 a
Immature survival (%)	80.00 ± 8.94 a	80.00 ± 8.95 a	85.00 ± 7.97 a	75.00 ± 9.67 a	75.00 ± 9.64 a
Male adult longevity (day)	5.50 ± 0.32 a	3.62 ± 0.36 b	3.12 ± 0.22 b	4.00 ± 0.31 ab	5.50 ± 1.15 a
Female adult longevity (day)	6.00 ± 0.32 a	3.87 ± 0.22 b	3.88 ± 0.19 b	3.9 ± 0.22 b	3.54 ± 0.15 b
Fecundity (egg per female)	88.00 ± 3.89 a	30.87 ± 1.63 c	19.05 ± 0.83 d	40.00 ± 3.42 b	40.27 ± 2.03 b
TPOP (day)	24.50 ± 0.26 b	24.12 ± 0.12 b	24.44 ± 0.17 b	24.60 ± 0.15 b	27.00 ± 0.13 a

TPOP: Total pre-ovipositional period. Means within rows followed by different lowercase letters indicate statistically significant differences (paired bootstrap test, α = 0.05).

**Table 4:** Life-table parameters (mean ± SE) of *Callosobruchus maculatus* treated with *Satureja hortensis* and *S. khuzistanica* essential oils and on untreated control group.

Parameter	Control	*S. hortensis*		*S. khuzistanica*	
		LC_30_	LC_50_	LC_30_	LC_50_
*r* (day^−1^)	0.130 ± 0.011 a	0.095 ± 0.011 bc	0.080 ± 0.010 c	0.112 ± 0.009 ab	0.106 ± 0.007 b
*R*_0_ (offspring)	35.20 ± 9.76 a	12.35 ± 3.44 bc	8.57 ± 2.14 c	20.00 ± 4.75 ab	22.15 ± 4.57 ab
*GRR* (offspring)	58.06 ± 12.65 a	17.50 ± 4.66 c	15.32 ± 3.21 c	30.38 ± 6.37 b	33.89 ± 6.57 b
*λ* (day^−1^)	1.13 ± 0.01 a	1.10 ± 0.01 bc	1.08 ± 0.01 c	1.11 ± 0.01 ab	1.11 ± 0.008 ab
*T* (day)	27.37 ± 0.34 b	26.24 ± 0.17 c	26.60 ± 0.23 bc	26.64 ± 0.17 bc	28.95 ± 0.19 a

Mean values in each row followed by different letters are significantly different according to paired-bootstrap test. (*p* < 0.05). *r*: Intrinsic rate of population increase, *R*_0_: Net reproductive rate, *GRR*: Gross reproductive rate, *λ*: Finite rate of increase, and *T*: Mean generation time.

## Data Availability

The original contributions presented in this study are included in the article. Further inquiries can be directed to the corresponding authors.
